# Isolation and identification of endophytic actinobacteria from *Iris persica* and *Echium amoenum* plants and investigation of their effects on germination and growth of wheat plant

**DOI:** 10.1002/fsn3.3488

**Published:** 2023-06-27

**Authors:** Hakimeh Oloumi, Moj Khaleghi, Ava Dalvand

**Affiliations:** ^1^ Department of Ecology, Institute of Science and High Technology and Environmental Sciences Graduate University of Advanced Technology Kerman Iran; ^2^ Department of Biology, Faculty of Sciences Shahid Bahonar University of Kerman Kerman Iran

**Keywords:** antioxidant activity, Endophytic *Actinobacteria*, salinity stress, *Streptomyces*, wheat plant

## Abstract

Plant biotechnology helps to develop different types of new products with increased resistance to disease, greater tolerance to drought and salt stress, and better nutritional value. The interaction of plants and microorganisms will play a significant role to achieve this purpose. The aims of this study were to isolate endophyte *Actinobacteria* strains of some medicinal plants and the investigation of their bioactive potential. 15 *Actinobacteria* strains were selectively isolated from *Persian iris* and *Echium amoenum* plants, and then their belonging to *Actinobacteria* phylum was confirmed using an *Actinobacteria*‐specific primer pair. The antioxidant activity of the crude extract obtained from the isolated strains was investigated based on DPPH method. Investigating the antioxidant activity of the crude extract showed that at a concentration of 100 μg/mL, the two strains EG1 and EG2 had 71% and 78% antioxidant activity, respectively. According to the phylogeny studies, it was determined that two strains belonged to the *Streptomyces* genus. The effect of supernatant achieved from selected endophytic strain on 35‐day wheat plants showed that the supernatant considerably promotes root and shoot growth and chlorophyll content under salinity stress (150 mM NaCl). In general, it can be concluded strains that live symbiotically with medicinal plants are rich sources of bioactive compounds. Therefore, identification of the bioactive compounds in the extract of isolated *Actinobacteria* from medicinal plants and further studies on their metabolism are suggested.

## Graphical abstract

Actinobacteria strains were selectively isolated from *Persian iris* and *Echium amoenum* plants, and then their belonging to Actinobacteria phylum was confirmed using an Actinobacteria‐specific primer pair. The effect of supernatant achieved from selected endophytic strain on 35‐day wheat plants showed that the supernatant considerably promotes root and shoot growth and chlorophyll content under salinity stress (150 mM NaCl).

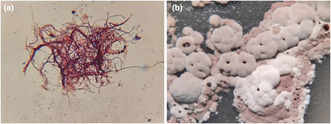

## 1 INTRODUCTION

During their growth, plants are constantly in contact with soil microorganisms, and in the meantime, some microorganisms create a symbiotic relationship with plants, plant–microorganism interaction have some beneficial effects such as accelerating plant growth (Ghodhbane‐Gtari et al., 2019). However, endophytic microorganisms living in plant tissues such as branches, leaves, roots, stems, and bark of plants have received much attention in recent years due to the wide range of secondary metabolites they produce (Zheng et al., 2016). Endophytes are microorganisms in plants that coexist with plants without harming them. Among all the symbiotic microorganisms, *Actinobacteria* have a great contribution in this interaction, *Actinobacteria* is one of the important groups of bacteria known as plant growth‐promoting bacteria (PGPB) due to their high distribution in the soil (10^8^–10^9^ per g) (Hamedi & Mohammadipanah, 2015). These bacteria are an important part of the soil microbial community and their uniqueness as producers of bioactive compounds has been well defined in the past years. The study of endophytic *Actinobacteria* leads to the isolation of new bioactive compounds having therapeutic, agricultural, and industrial properties (Nafis, Elhidar, et al., 2018; Nalini & Prakash, 2017).

Biological interactions between plants and endophytic microorganisms may cause reduced amounts of biological stressors. In fact, metabolic studies have shown that endophytic microorganisms play a role in strengthening and growing plants by stimulating the production of plant hormones such as auxins and gibberellins, or as plant protection agents against microbial pathogens and insects (Ek‐Ramos et al., 2019; Oubaha et al., 2019). In a research conducted in 2014, it was observed that the overexpression of malic acid‐producing genes in the endophytic bacterial strains increased the release of phosphate in the soil, and as a result led to higher number of lateral branches with higher length of the stem and roots in wheat plants (Jog et al., 2014). In the research of Yandigeri et al., 2012 46 endophyte *Actinobacteria* strains were isolated from the roots of drought‐resistant plants. Their results also showed that these bacterial strains had the ability to increase plant growth and increase drought tolerance. In molecular identification, the top three strains were confirmed as *Streptomyces* sp (Yandigeri et al., 2012).

In recent years, the attention of researchers has been drawn to endophytic microorganisms as producers of bioactive compounds useful for various industries, manifesting the importance of this group of microorganisms. Almost all plant species can be resources of endophytic microorganisms and are considered as promising sources of new biological compounds. *Persian iris* (*Iris persica*) and *Echium Amoenum* were selected in this study to isolate their endophytic *Actinobacteria* strains. In this project, the standard DPPH assay was used to determine antioxidant activity of isolated *Actinobacteria* extracts. The role of endophytic bacteria extract was studied on plants under salinity stress. For this purpose, effects of the bioactive compounds extracted from selected endophyte strains were studied on the root and shoot growth, total chlorophyll amounts and relative water content of wheat seedlings under salinity stress.

## 2 MATERIALS AND METHODS

### 2.1 Collection of plant samples

Plant parts of two medicinal plants iris (*Iris persica*) and *Echium amoenum*, were collected from Jiroft city, Kerman province (57 49 14/591E, 28 53 51/735 N) (57 28 26/168E, 29 11 21/098 N). In order to isolate endophytic *Actinobacteria* plant samples stored in sterile containers and transported to the laboratory as soon as possible. The isolation of strains was done from the roots, stems and leaves of the plants. For this purpose, different plant organs including leaves, shoots and roots were sterilized with 70% ethanol for 5 min and 3% sodium hypochlorite solution for 20 min. Samples were then washed with sterile distilled water for three times, and from washing water transferred to the NA (Nutrient agar: Peptone 5 g/L, Meat extract 3 g/L, Agar 15 g/L) culture medium to ensure the absence of microbial and fungal contaminations (Verma et al., 2009).

### 2.2 Selective isolation of endophytic *Actinobacteria*

The specific growth media of *Actinobacteria*, including ISP2 (yeast extract 4 g/L, malt extract 10 g/L, dextrose 4 g/L, distilled water 1L), ISP4 (soluble starch 10 g/L, MgSO_4_ 7H_2_O 1 g/L, NaCl 1 g/L, (NH_4_)_2_SO_4_ 2 g/L, CaCO_3_ 2 g/L, trace salt solution 1 mL, distilled water 1 L), AIA (actinomycete isolation agar: sodium caseinate 2 g/L, L‐asparagine 0.1 g/L, sodium propionate 4 g/L, dipotassium phosphate 0.5 g/L, magnesium sulphate 0.1 g/L, ferrous sulphate 0.001 g/L, agar 15 g/L, distilled water 1L), gauze (Soluble Starch 20 g/L, KNO_3_ 1 g/L, NaCl 0.5 g/L, MgSO_4_ 7H_2_O 0.5 g/L, K_2_HPO_4_ 0.5 g/L, FeSO_4_ 7H_2_O 0.01 g/L, distilled water 1L), ISP3 (Oatmeal 20 g/L, trace salt solution 1 mL, distilled water 1L), SCA (starch casein Agar: soluble starch 10 g/L, casein 0.3 g/L, KNO_3_ 2 g/L_,_ MgSO_4_.7H_2_O 0.05 g/L, K_2_HPO_4_ 2 g/L, NaCl 2 g/L, CaCO_3_ 0.02 g/L, FeSO_4_ 7H_2_O 0.01 g/L, distilled water 1 L), containing nalidixic acid (10 μg/mL) and nystatin (50 μg/mL) were used to isolate *Actinobacteria* endophyte strains from plant tissues. Isolated *Actinobacteria* incubated for 21 days at 30°C. After the incubation period, colonies with chalky and powdery morphology were isolated and purified stored in 20% glycerol at −20°C for further studies (Ranjan & Jadeja, 2017). Visual observations of morphological and microscopic features were also performed using a stereomicroscope light microscope and gram staining.

### 2.3 DNA extraction and molecular identification

To determine the phylogenetic characteristics, the genomic DNA of strains was extracted using the Bacterial DNA isolation kit (DENA Zist). Polymerase chain reaction (PCR) for the 16SrRNA gene was performed from the DNA samples extracted as follows: initial denaturation for 5 min at 95°C, denaturation at 94°C with 35 cycles for 1 min, annealing at 56°C for 1.5 min, extension at 72°C for 2 min, and final extension for 10 min at 72°C (Musa et al., 2020).

The belonging of the strains to *Actinobacteria* phylum was validated with the help of *Actinobacteria* specific primer pair S‐C‐Act‐235 (5’‐CGCGGCCTATCAGCTTGTTG‐3′), S‐C‐Act‐878 (5′‐ CCGTACTCCCCAGGCGGGG −3′). The universal primer pair 27F (5’ AGAGTTTGATCCTGGCTCAG‐3′) and 1492 R (5’‐GGTTACCTTGTTACGACTT‐3′) was used as a template for sequencing. The PCR products were purified using a PCR kit (Thermo Scientific) and sequenced by Macrogen (South Korea).

The similarity of the studied strains was checked using BLAST analysis (http://blast.ncbi.nlm.nih.gov/Blast.cgi). Alignment and drawing of phylogenic tree were done by MEGA X software and Bootstrap analysis with 1000 repetitions (Kumar et al., 2018).

### 2.4 Fermentation and extraction

*Actinobacteria* strains were incubated in 200 mL Gauze medium (soluble starch 20 g/L, KNO_3_ 1 g/L, NaCl 0.5 g/L, MgSO_4_ 7 H_2_O 0.5 g/L, K_2_HPO_4_ 0.5 g/L, FeSO_4_ 7 H_2_O 0.01 g/L, distilled water 1 L), for 7 days at 30°C and 140 rpm. Then, microbial suspension was centrifuged at 11,000 *g* and 4°C for 30 min, and the obtained supernatant was sterilized using a 22‐μm filter. The sterile supernatant was mixed with the organic solvent ethyl acetate (Merck) (1:1 (v/v)) and the organic phase was separated after 24 h, (Mohammadi et al., 2022). The obtained extract was dried and stored at −20°C.

### 2.5 Antioxidant assay

The free radical inhibition activity was measured using the DPPH (Sigma‐Aldrich) (1,1‐diphenyl‐2‐picrylhydrazyl) method, as a standard method for determining antioxidant power of extract. 100 μL of 0.1% DPPH solution was added to 100 μL of different concentrations (100, 80, 60, 40, 20, 10 μg/mL) of microbial extracts in a 96‐well microtiter plate. The plates were incubated at room temperature for 30 minutes in the dark, and the optical density was measured using a spectrophotometer at 515 nm (Dahal et al., 2020). The absorbance of the DPPH was noted as control (no extract/standard) and ascorbic acid was used as a positive control. The inhibition percentage was calculated using the following equation where 𝐴 defines absorbance of DPPH in control and 𝐵 is sample absorbance.

$$\text{Inhibitory Activity}\;\left(\%\right)=\text{[(34𝐴−34𝐴𝐵)/34𝐴𝐵𝐴]}\times 100$$

### 2.6 Endophytic supernatant treatment on wheat seedlings

In order to investigate the effect of microbial supernatant on plant growth, 25‐day‐old plants were used. Wheat seeds (*Triticum sativum* Amir) obtained from the Agricultural Research Center; Kerman, Iran, were cultivated in pots containing acid‐washed sandy soil under greenhouse conditions in a completely randomized design. One week after germination, the seedlings were irrigated with complete Hoagland’ nutrient solution. In the third week of seedling growth, the pots were divided into two groups of control and saline groups. On the control group, irrigation was done every other day with nutrient solution and on the salinity stressed group, irrigation was done with nutrient solution containing 150 mM sodium chloride. One week later, each group was again divided into two groups of supernatant or water spray group. Plants in these group were sprayed with the microbial supernatant or sterilized distilled water, every 2 days in between. The supernatant was obtained from isolated EG2 *Actinobacteria* strains with the highest antioxidant activity among all isolated strains. The supernatant or water was sprayed on seedling shoots until the liquid began to drip off from the leaves. After 10 days from the start of supernatant treatment, the plants were harvested to measure roots and shoots length. The extended leaf of each plant was used to measure relative water content and total chlorophyll. The total chlorophyll content was measured using a portable chlorophyll meter and the leaf water content was measured based on method Mullan and Pietragalla (2012) (Mullan & Pietragalla, 2012). To measure RWC, leaf fresh weight, turgid weight (held 5 hours in deionized water), and the leaf dry weight (72 h at 70°C) were recorded for the extended leaf of harvested seedlings. Relative water content was calculated using following equation:

$$\frac{\text{Fresh Weight}-\mathrm{Dry}\;\text{Weight}}{\text{Torgor weight}-\mathrm{Dry}\;\text{weight}}\times 100$$

### 2.7 Statistical analysis

All experiments were performed with three replications. The results were analyzed using SPSS 21 software and one way ANOVA. To compare means of groups, Duncan’ test was carried out with *p* value ≥.05.

## 3 RESULTS

### 3.1 Isolation and identification of *Actinobacteria*

**FIGURE 1:**
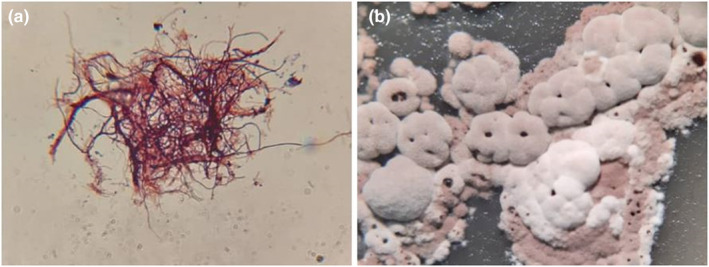
EG2 strain isolated from *Echium amoenum* roots. Morphology of colony (a), morphology of bacteria (b).

**FIGURE 2:**
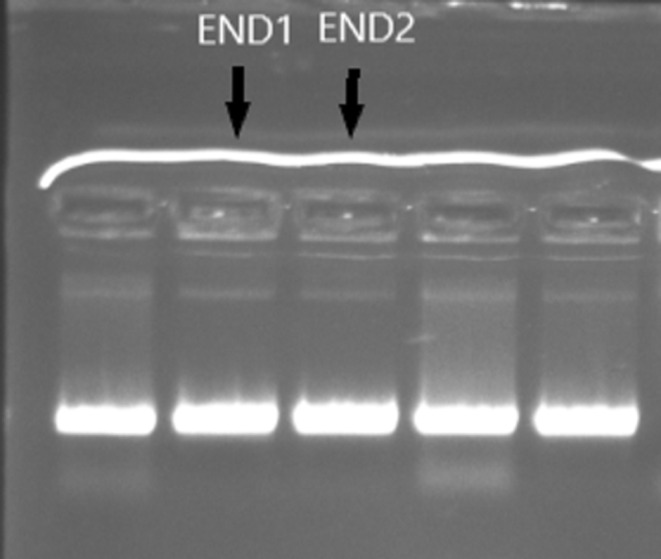
Image of 600‐bp band of *Actinobacteria* phylum‐specific primer.

In this research, *Actinobacteria* strains were isolated from Persian iris and borage. Totally, 15 strains of *Actinobacteria* were isolated based on colony morphology. Powdery and cottony colonies were selected (Figure 1a) and Gram's method staining was done for *Actinobacteria* identification. The staining results confirmed the presence of Gram‐positive filamentous bacteria (Figure 1b). Two *Actinobacteria*‐specific primes including S‐C‐Act‐235(5’‐CGCGGCCTATCAGCTTGTTG‐3′), and S‐C‐Act‐878(5’‐CCGTACTCCCCAGGCGGGG‐3′), were used for PCR analysis. Results showed the presence of a 600‐bp single band (Figure 2) which confirms that the isolates belonged to *Actinobacteria phylum*. Based on these results, 9 strains among 15 isolated strains belonged to *E. amoenum* plants (7 strains from the root and 2 strains from the leaves), and 6 of them have been extracted from *Persian Iris* (3 strains from the root, 2 strains from the leaves, and 1 strain from the stem). In Table 1 shows the characteristics of the colony morphology of the isolates are given.

**TABLE 1:** Colony morphology of endophyte strains isolated from *Echium amoenum* and *Iris persica* plants.

Strain	Aerial mycelium color	Substrate mycelium color	Pigment color	Plant	Organ
EG1	Gray	White	Brown	*E. amoenum*	Root
EG2	White	White	Purple	*E. amoenum*	Root
EG3	White	White	Yellow	*E. amoenum*	Root
EG4	Red‐Black	Yellow	Black	*E. amoenum*	Leaf
EG5	White	White	–	*E. amoenum*	Root
EG6	White‐Yellow	White	–	*E. amoenum*	Root
EG7	White	White	White‐yellow	*E. amoenum*	Root
EG8	Green‐Gray	Brown	Green‐black	*E. amoenum*	Leaf
EG9	White‐Gray	Yellow	Gray	*I. persica*	Root
EG10	White	Brown	–	*I. persica*	Stem
EG11	White	Gray	Green	*I. persica*	Root
EG12	Gray	Brown	Brown	*I. persica*	Root
EG13	Gray	White	Brown	*I. persica*	Leaf
EG14	White	White	Creamy	*I. persica*	Leaf
EG15	White	Colorless	Creamy	*I. persica*	Root

### 3.2 Antioxidant assay

**FIGURE 3:**
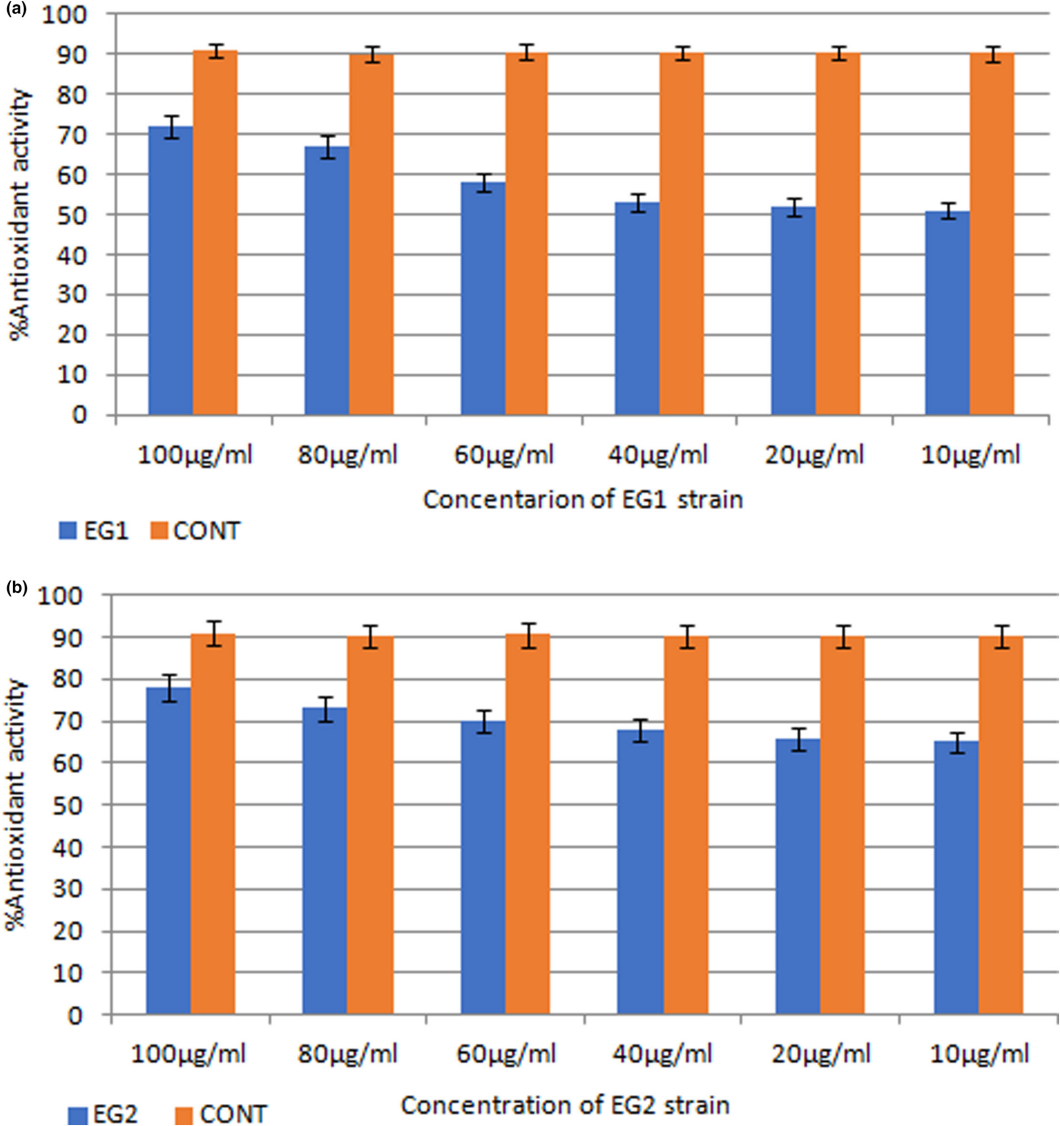
Antioxidant effects of *Actinobacteria* strains based on DPPH method, (a) the antioxidant activity of different concentrations of EG1 strain extracts and (b) EG2 strain.

All 15 strains were investigated for their antioxidant activity based on DPPH method. Our results showed that strains EG1 and EG2 isolated from *E. amoenum* root plant had the highest antioxidant activity (Figure 3). According to the obtained results, the EG2 strain at a concentration of 100 μg/mL has 78% antioxidant activity and the EG1 strain at the same concentration has 71% antioxidant effect, since ascorbic acid (Sigma‐Aldrich) as a control had 91% antioxidant activity at 100 μg/mL. Results also showed that the antioxidant activity of both strains was dose‐dependent and it promoted with increasing concentration (Figure 3).

### 3.3 Identification of EG1, EG2 strains

**FIGURE 4:**
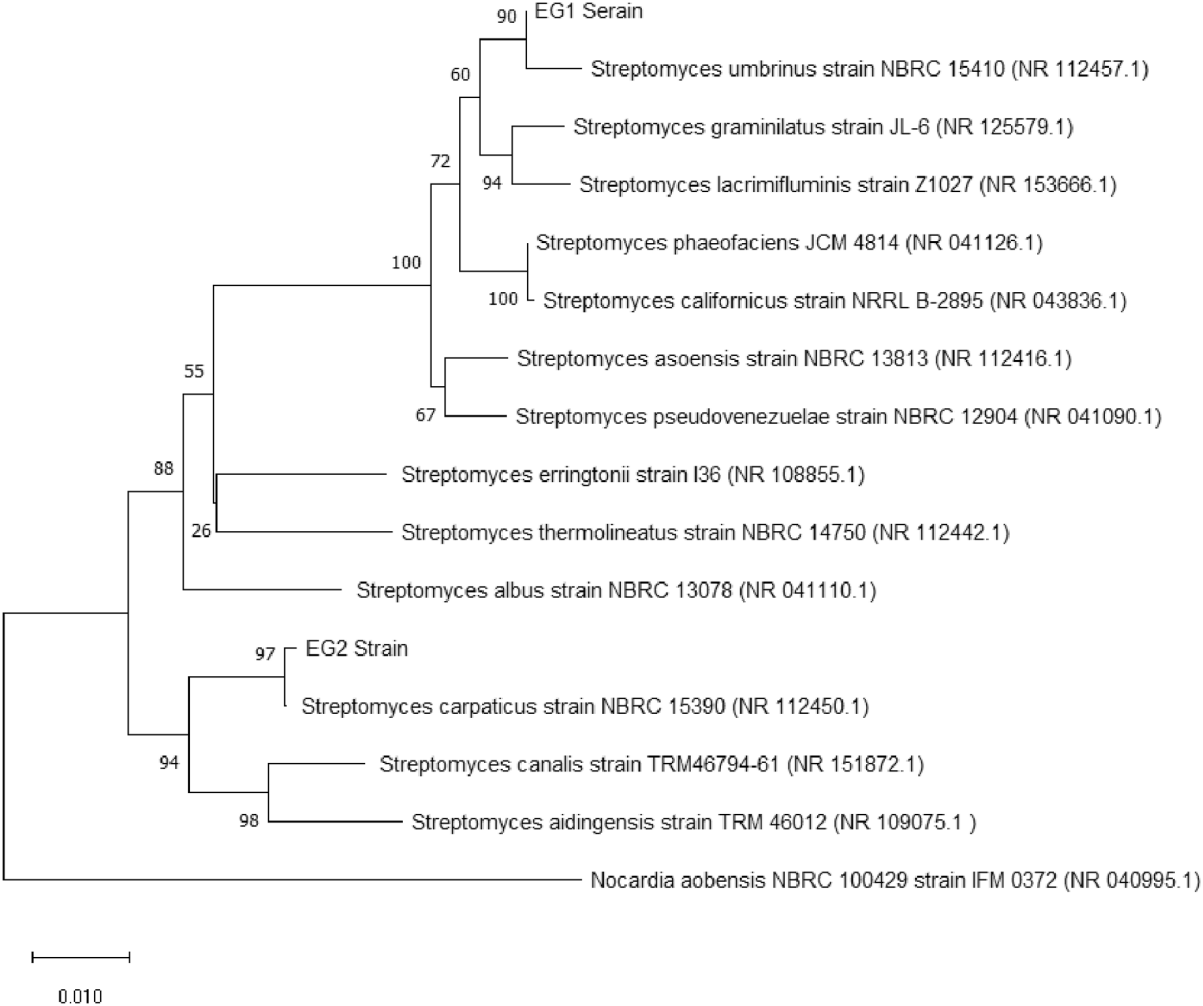
The neighbor‐joining tree based on the 16S rRNA sequences of selected strains shows the relationship between EG1, EG2 strain, and other related taxa. The numbers at the nodes indicate the levels of bootstrap support based on 1000 resampled data sets. *Nocardia aobensis* NBRC 100429 strain IFM 0372 (NR 040995.1) was used as an out‐group.

Based on the sequence analysis of 16S rRNA, the top two strains of this study were belonged to the genus *Streptomyces*. According to the results, strain EG1 with more than 99% similarity was identified as *Streptomyces umbrinus strain* NBRC 15410 and strain EG2 with more than 99% similarity as *Streptomyces carpaticus* strain NBRC 15390 (Figure 4).

### 3.4 The supernatant effects on growth and physiological parameters of wheat seedlings

**FIGURE 5:**
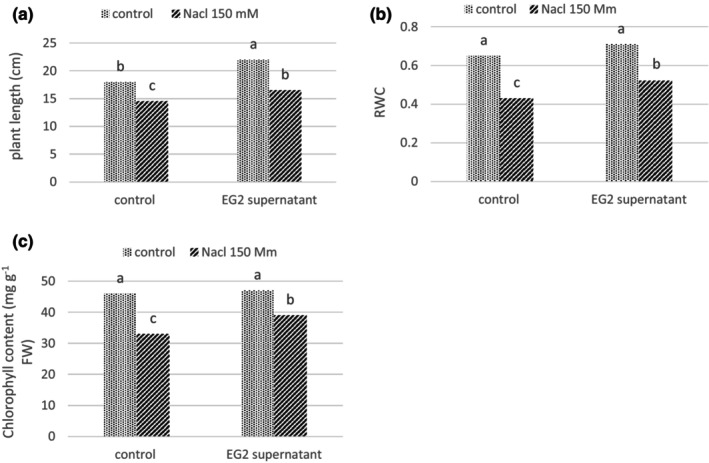
The effect of EG2 supernatant on wheat plants under NaCl (150 mM) stress. (a) Plant length, (b) relative water content (RWC), (c) chlorophyll content. Columns with the same letters do not show significant differences based on Duncan's test (*p* ≤ .05).

In this study, the effect of microbial supernatant from EG2 strains was investigated on wheat plants under salinity stress. Our results showed that the foliar spray of supernatant improved the growth and physiological parameters of wheat seedlings under both salt stress and nonstress conditions (Figure 5).

Based on the morphological analysis, our results showed that the longitudinal growth of roots and shoots were significantly (*p* < .05) increased in supernatant treated wheat plants grown under natural and salinity conditions (Figure 5a). The results also showed that the relative water content of leaves was improved by up to 20% in plants under salt stress treated with the *Actinobacteria* supernatant comparing to salt‐stressed seedlings (Figure 5b). Under salinity stress, total chlorophyll content increased by 15% in supernatant treated seedlings, but supernatant had no significant effect on chlorophyll content of plants grown under control condition (no salinity, Figure 5c).

## 4 DISCUSSION

Endophytic *Actinobacteria* has been shown as potentially rich sources of metabolites which give them ability of promoting the growth of their host plant. These metabolites also lead to the improvement of the disease symptoms caused by plant pathogens or various environmental stresses (Golinska et al., 2015).

In this study, we investigated the ability of endophytic *Actinobacteria* isolated from two medicinal plants collected from their natural habitants in south of Iran. Different parts of *E. amoenum* and iris plants were gathered and transported into laboratory for further studies and isolation of their *Actinobacteria* strains. *Actinobacteria*‐specific culture media such as ISP2, ISP3, ISP4, Gauze, SCA, AIA, were used for isolation of *Actinobacteria* strains. Colonies of *Actinobacteria* appeared on the agar surface after 2 weeks. Among all colonies, 15 powdery and dried colonies were isolated and purified as *Actinobacteria* strains.

The antioxidative activities of identified strains were also investigated. Among 15 investigated strains, two strains showed the best results, as EG1 and EG2 at 100 μg/mL, inhibited more than 70% of free radicals, which according to previous researches on *Actinobacteria* isolates from various sources is a desirable result (Janardhan et al., 2014). In research conducted by Siddharth & Vittal, 2018, the studied *Actinobacteria* strain was able to inhibit 56% of free radicals at a concentration of 1 mg/mL. As it was observed from our results, the strains EG1 and EG2 in this study at 0.1 mg/mL, showed more than 70% inhibitory effects on DPPH radicals, which is significantly high compared to previous studies (Siddharth & Vittal, 2018). We also observed that the antioxidant power of the *Actinobacteria* strains reinforce with increasing the supernatant concentration. The obtained results from (Karthik et al., 2013) research also confirm the dose‐dependent antioxidative effects of *Actinobacteria*. Results from 16S rRNA analysis showed that selected strains EG1 and EG2 were belonging to *Streptomyces* species. Studies have shown that *Streptomyces* species of *Actinobacteria* phylum as endophytes, are able to communicate with various types of plants (Verma et al., 2009), In the study of Nafis et al., the NAF‐1 strain isolated from medicinal plant *Aloe vera*, having various biological properties and antioxidant activity, was identified as a strain of the genus *Streptomyces* (Nafis et al., 2018b).

*Streptomyces* species are known as important bacterial source of secondary metabolites (Takahashi & Nakashima, 2018), therefore, their performance as the superior strains of this study, especially the EG1 strain, was not far from expectation. Similar to the results of present study, Nafis et al., reported that the strain AS25 isolated from the rhizosphere soil of *Alyssum spinosum* belonging to the genus *Streptomyces*, is a producer of some important bioactive compounds (Nafis, Oubaha, et al., 2018). Many researchers recommend the application of *Actinobacteria* as effective and stimulator factor in plant growth promotion. These studies show that plant growth‐stimulating microorganisms are rich sources of valuable secondary metabolites which can be used in sustainable agriculture (Pellegrini et al., 2020).

Our results showed that the supernatant obtained from EG2 *Actinobacteria* strain would improve wheat plant growth under normal and stress conditions. Therefore, significant improvement of wheat plants growth under normal and stress conditions by *Actinobacteria* supernatant can be associated to the nutritional enrichment of supernatant which is absorbed by plant leaf surface.

Salt stress cause a wide range of damaging symptoms on plants, such as inhibition of shoot and root growth, imbalance of ion homeostasis, and metabolic changes (carbon and metabolism, photosynthetic pigments disruption induction of oxidative stress) (Xu et al., 2016). Salinity condition considerably reduced wheat plant growth, relative water content, and total chlorophyll content of leaves. In this project, however, the improved growth of wheat plant after supernatant treatment was accompanied with higher chlorophyll content and relative water content in salt‐stressed plants. Similar to our results, Zahra et al. (2020) reported growth retardation of sunflower plants under salinity. In their experiments using endophytic *Actinobacteria* of a halophytic desert plant, *Pteropyrum olivieri*, they show that endophytic *Actinobacteria* improve growth of sunflower plants through modulation of protein and chlorophyll content (Zahra et al., 2020). Passari et al also reported that tomato plants inoculated with *Streptomyces thermocarboxydus* strain BPSAC147 show disease resistance and improved growth through the positive effect on the electron transport rate (ETR) of PSII, and the process of photosynthesis, and enhanced chlorophyll fluorescence achieved after inoculation (Passari et al., 2019). However, there is lack of information about the mode of action of endophytic *Actinobacteria* on chlorophyll content in plants. It seems that *Actinobacteria* having antioxidative properties protect chlorophyll against degradation in salt stress conditions.

Plants’ tolerance to salt stress is also associated with the modulation of antioxidant enzymes. Antioxidant defense systems are involved in the removal of ROS either by antioxidant enzyme activities or production of antioxidant compounds such as glutathione and ascorbic acid (Rouhier et al., 2008). In barley plants, it was reported by Sarma et al. (2011) that *Piriformospora indica*, an endophytic bacteria induces salt tolerance by increasing the levels of antioxidants (Sarma et al., 2011). As we observed in this project, the EG2 strain extract has a powerful antioxidant capacity, based on DPPH method. Therefore, plant growth improvement in salt‐stressed wheat plants treated by EG2 supernatant may be attributed to its antioxidant compounds.

The present study suggests that the endophytic *Actinobacteria* isolated from *E. amoenum* plants can be used as an agent to control salinity stress in *T. sativum* plants, for their high antioxidant capacity. However, more complementary researches are still needed about their gene expression and physiological and biochemical impacts of *Actinobacteria* on plants under stress.

## AUTHOR CONTRIBUTIONS

**Hakimeh Oloumi:** Conceptualization (equal); formal analysis (equal); funding acquisition (equal); investigation (equal); methodology (equal); project administration (equal); supervision (equal); validation (equal); visualization (equal); writing – original draft (equal); writing – review and editing (equal). **Moj Khaleghi:** Methodology (equal); resources (equal); software (equal); validation (equal); visualization (equal); writing – original draft (equal). **Ava Dalvand:** Formal analysis (equal); investigation (equal); software (equal); writing – original draft (equal).

## CONFLICT OF INTEREST STATEMENT

All authors declared that they have no conflict of interest.

## Data Availability

The data that support the findings of this study are available from the corresponding author upon reasonable request.
